# An Exploration of Sexual Agency in the Aging Population

**DOI:** 10.1093/geroni/igaa057.2034

**Published:** 2020-12-16

**Authors:** Lilanta Bradley

**Affiliations:** University of Alabama, Tuscaloosa, Alabama, United States

## Abstract

There are some unique social and environmental challenges to geriatric sexual well-being. This presentation explores sexual agency in the aging population with a special emphasis on barriers and facilitators of sexual agency in long-term care settings. We will situate late-life sexuality literature with Cense’s (2019) theory of sexual agency. Her four components of moral agency, embodied agency, narrative agency, and bonded agency will be used to examine the older person’s perceptions, beliefs, and behaviors associated with sexual autonomy from a macro and micro lens. This framework uses assertions that agency is built on self-concepts from the individual and relational level; reflects power dynamics and social inequalities; and requires strategic negotiation of social norms, policies, and relationships to shape one’s autonomy and sexual self perceptions. We will also discuss gaps in the literature regarding gender, race/ethnicity, and geographical differences that can impact an older person’s sexual agency and overall sexual well-being.

